# Phase I study of amatuximab, a novel monoclonal antibody to mesothelin, in Japanese patients with advanced solid tumors

**DOI:** 10.1007/s10637-014-0196-0

**Published:** 2014-12-12

**Authors:** Yasuhito Fujisaka, Takayasu Kurata, Kaoru Tanaka, Toshihiro Kudo, Kunio Okamoto, Junji Tsurutani, Hiroyasu Kaneda, Isamu Okamoto, Masayuki Namiki, Chifumi Kitamura, Kazuhiko Nakagawa

**Affiliations:** 1 Department of Medical Oncology, Kinki University Faculty of Medicine, 377-2 Ohnohigashi, Osakasayama, Osaka 589-8511 Japan; 2 Eisai Co., Ltd., Koishikawa 4-6-10, Bunkyo-ku, Tokyo 112-8088 Japan; 3 Present Address: Clinical Research Center, Osaka Medical College Hospital, 2-7 Daigakumachi, Takatsuki, Osaka 569-8686 Japan; 4 Present Address: Department of Thoracic Oncology, Kansai Medical University Hirakata Hospital, 2-3-1 Shin-machi, Hirakata, Osaka 573-1191 Japan; 5 Present Address: Department of Frontier Science for Cancer and Chemotherapy, Graduate School of Medicine, Osaka University, E21-19, 2-2 Yamadaoka, Suita, Osaka 565-0871 Japan; 6 Present Address: Department of Medical Oncology, Kishiwada Municipal Hospital, 1001 Gakuhara-cho, Kishiwada, Osaka 596-8501 Japan; 7 Present Address: Center for Clinical and Translational Research, Kyushu University Hospital, 3-1-1 Maidashi, Higashi-ku, Fukuoka 812-8582 Japan

**Keywords:** Amatuximab, Antibody, Mesothelin, MORAb-009, Phase I study

## Abstract

Amatuximab is a chimeric monoclonal antibody that targets mesothelin, which is expressed in virtually all mesotheliomas and pancreatic adenocarcinomas. The objective of this study was to determine the dose-limiting toxicity and the maximum tolerated dose. Patients with mesothelioma, pancreatic adenocarcinoma or other mesothelin-positive solid tumors were eligible for this study. Amatuximab was administered weekly as an intravenous infusion in 4-week cycles at progressively increasing doses ranging from 50 to 200 mg/m^2^. Seventeen patients received amatuximab. Two dose-limiting toxicities were observed: one at 50 mg/m^2^ and one at 200 mg/m^2^; the maximum tolerated dose of this study was determined to be 200 mg/m^2^. Of the 17 patients, 13 patients (76.5 %) experienced treatment-related adverse events. The most common adverse events were grade 1 fatigue (29.4 %) and pyrexia (23.5 %). The maximum serum concentration and area under the concentration curve values increased in an almost dose-proportional manner. Three patients had stable disease. Amatuximab was generally well tolerated at doses up to 200 mg/m^2^. The pharmacokinetic profile of amatuximab in the Japanese population was similar to that seen in the United States population (Clinical Trials.gov Identifier: NCT01018784).

## Introduction

Amatuximab (MORAb-009) is a high affinity, chimeric, IgG1/κ monoclonal antibody targeting mesothelin, which is a glycosyl-phosphatidyl inositol-linked membrane glycoprotein. The normal biological function of mesothelin is not known. Mesothelin does not appear to be required for either normal development or reproduction since there were no apparent abnormalities in mesothelin knockout mice [1]. However, recent studies have shown that mesothelin binds to the cell surface mucin, MUC16 (CA-125) [2–4], which is commonly used as a marker to follow patients with ovarian cancer. This interaction is thought to mediate cell adhesion, suggesting a role in cancer metastasis. Mesothelin is highly expressed in many common epithelial malignancies; however, the expression in normal tissues is limited to mesothelial cells lining the pleura, pericardium, and peritoneum [5]. Immunohistochemistry studies have shown that mesothelin is expressed in virtually all mesotheliomas and pancreatic ductal adenocarcinomas, a high percentage of epithelial ovarian cancers, and non-small cell carcinomas of the lung [6–10]. In addition, mesothelin is expressed to varying degrees by other tumors including endometrial adenocarcinoma, gastric adenocarcinoma, and cholangiocarcinoma [11].

In vitro*,* amatuximab elicits antibody-dependent cellular cytotoxicity against mesothelin-expressing tumor cell lines [12]. In vivo*,* tumor xenograft studies showed that amatuximab plus chemotherapy led to a greater reduction in the growth of mesothelin-expressing tumors than either amatuximab or chemotherapy alone [12].

In the previous United States (US) phase I study, the single-agent maximum tolerated dose (MTD) of amatuximab was 200 mg/m^2^. Two dose-limiting toxicities (DLTs) were noted at the 400 mg/m^2^ dose level [13].

On the basis of these pre-clinical and clinical results, we conducted a phase I study of amatuximab in Japanese patients with solid tumors. The primary objective of this study was to determine DLT and MTD in Japanese patients, and key secondary objectives were to examine the pharmacokinetics, anti-amatuximab antibody formation (human anti-chimeric antibody: HACA), mesothelin expression, and preliminary antitumor effect of amatuximab.

## Materials and methods

### Study design

This was a phase I dose-escalation study (MORAb-009-J081-102 study) designed to determine DLT and MTD in Japanese patients with solid tumors.

Amatuximab was administered weekly as an intravenous infusion in 4-week cycles until disease progression or the occurrence of a DLT. The first infusion was started at a rate of 1 mg/min. If no allergic reactions occurred within 30 min, the infusion rate could be increased up to a maximum rate of 5 mg/min. The second infusion could be started at the rate tolerated in the prior infusion. Initially, prophylactic premedication for allergic reactions was prohibited. During the course of the study, the protocol was amended to require premedication of antihistamines and acetaminophen prior to all infusions. This occurred after the fourth patient was dosed at 50 mg/m^2^.

In this study, the standard 3 + 3 dose escalation design was used and intra-patient dose escalation was not allowed. The starting dose of 50 mg/m^2^ was chosen due to the occurrence of a single DLT (deep venous thrombosis) in the US phase I study at the 100 mg/m^2^ dose [13]. The protocol specified that if a DLT occurred in the first three patients receiving either 50 or 100 mg/m^2^ dose, an additional three patients would be treated at this same dose level. If no additional DLTs occurred, then an escalation to the next dose could proceed. Six patients were required at the 200 mg/m^2^ dose level even if no DLTs were observed, as the dose was the MTD of the US phase I study. The highest tolerated dose was defined as MTD of this study. If two or more patients developed a DLT at a given dose, the prior lower dose was declared as the MTD. The following treatment-related toxicities that occurred in cycle 1 were defined as DLTs: any grade 4 hematologic toxicity except for a grade 4 leukopenia or neutropenia (grade 4 leukopenia or neutropenia had to persist for longer than 7 days to be defined as a DLT); any grade 3 or higher neutropenia with fever of ≥38.0 °C; any grade 3 thrombocytopenia requiring blood transfusion; any grade 3 gastrointestinal toxicities (except for nausea, vomiting, or diarrhea that was controllable by an antiemetic or antidiarrheal agent); and any grade 3 non-hematologic toxicity with the exception of abnormal laboratory parameters not requiring treatment.

### Patients and eligibility criteria

Japanese patients with histologically or cytologically diagnosed solid tumors not responsive to standard therapy or lacking appropriate treatment options were eligible for this study. In addition, the patients’ tumor was required to be mesothelin positive as confirmed by immunohistochemistry (IHC), except for patients with either a mesothelioma or a pancreatic adenocarcinoma as mesothelin has been reported to be expressed in virtually all of these types of tumors [6–8]. Other key inclusion criteria were as follows: age of 20–79 years; a life expectancy of 12 weeks or more; Eastern Cooperative Oncology Group performance score of 0 or 1; adequate organ function [hemoglobin ≥9.0 g/dL; neutrophil count ≥1.5 × 10^3^/μL; white blood cell count 3.0–12.0 × 10^3^/μL; platelet count ≥10 × 10^4^/μL; aspartate transaminase (AST) ≤5 × upper limit of normal (ULN); alanine transaminase (ALT) ≤5 × ULN; alkaline phosphatase ≤5 × ULN; total bilirubin ≤2.0 mg/dL; serum creatinine ≤2.0 mg/dL]. Patients were excluded from the study if they met any of the following criteria: having a brain metastasis presenting with clinical symptoms or requiring medical treatment; being positive for human immunodeficiency virus antibody, hepatitis C virus antibody, or hepatitis B surface antigen; severe systemic infection requiring treatment; history of hypersensitivity to protein formulation including monoclonal antibody, other active malignancies; body cavity fluids (such as pleural effusion or ascites) that could not be controlled by drainage.

### Safety assessments

Adverse events (AEs) were graded based on the Common Terminology Criteria for Adverse Events (CTCAE) v3.0, and were coded based on Medical Dictionary for Regulatory Activities v15.1.

Since amatuximab is a human-mouse chimeric monoclonal antibody, there is a potential for immune mediated AEs (e.g., allergic reaction). These AEs were classified as adverse events of interest (AEIs) based on the judgement by the investigators.

### Antitumor activity

Tumor assessment was performed every two cycles by investigators according to Response Evaluation Criteria in Solid Tumor v1.0 [14].

### Pharmacokinetic assessments

During cycle 1, blood samples were collected on day 1 (within 1 h before the start of the amatuximab infusion; 0.5 h after the start of the amatuximab infusion; at the end of the infusion; 0.5, 1, 2, and 4 h after the end of the amatuximab infusion; 24 and 72 h after the start of the amatuximab infusion); on day 8 and 15 (within 1 h before the start of the amatuximab infusion); and on day 22 (within 1 h before the start of the amatuximab infusion; 0.5 h after the start of the amatuximab infusion; at the end of the infusion; 0.5, 1, 2, and 4 h after the end of the amatuximab infusion; and 24 h after the start of the amatuximab infusion). In all subsequent cycles, blood samples were collected on day 1 (within 1 h before the start of amatuximab infusion) only. Serum amatuximab concentrations were measured with an electrochemiluminescence immunoassay (ECLIA) which has a lower limit of quantification of 0.098 μg/mL.

Serum concentrations of amatuximab were measured to determine standard pharmacokinetic parameters including maximum serum concentration (C_max_), area under the concentration curve (AUC), time of maximum concentration (t_max_), and terminal half-life (t_1/2_). Pharmacokinetic parameters were calculated by a noncompartmental approach using WinNonlin software v6.2 (Pharsight, Sunnyvale, CA).

### Human anti-chimeric antibody assessments

In cycle 1, blood samples were collected on day 1 and day 15 (within 1 h before the start of the amatuximab infusion). In cycle 2 and thereafter, blood samples were collected on day 1 (within 1 h before the start of amatuximab infusion). The qualitative serum HACA level was measured with ECLIA.

### Immunohistochemistry

Tumor tissue samples obtained from 53 patients were assayed to investigate mesothelin expression by IHC using a commercially available anti-mesothelin antibody, Novocastra Lyophilized Mouse Monoclonal Antibody Mesothelin (Leica Biosystems, Newcastle, UK). Immunoreactive intensities were graded as 0, no reaction; 1+, weak (i.e., stained light brown with the thin rim along the cell membrane); 2+, moderate (i.e., stained deep-brown with the thin rim along the cell membrane); and 3+, strong (i.e., stained deep-brown with the thick rim along the cell membrane or higher intensity). If any immunohistochemical stains were observed in tumor tissue samples, the patient was determined to be mesothelin positive by a pathologist.

## Results

### Patient characteristics

Of 58 screened patients, 17 patients were enrolled between November 2009 and March 2012 at Kinki University Faculty of Medicine in Japan. The median age of the patients was 62 years (range, 56–79 years) (Table 1). The performance status of all patients were 0 or 1. Of 17 patients, the most common tumor types were colorectal cancer (seven patients) and pancreatic adenocarcinoma (six patients).

**Table 1:** Patient characteristics

Characteristic	No. of patients (*n* = 17)
Age (years)	
Median (Range)	62 (56–79)
Sex (*n*)	
Male	12
Female	5
Body surface area (m^2^)	
Median (Range)	1.64 (1.18–1.88)
ECOG performance status (*n*)	
0	10
1	7
Tumor type (*n*)	
Colorectal cancer	7
Pancreatic adenocarcinoma	6
Head and neck cancer	2
Mesothelioma	2

*ECOG* Eastern Cooperative Oncology Group

### Dose of amatuximab

All 17 patients were treated with amatuximab at one of the dose levels from 50 mg/m^2^ to 200 mg/m^2^ according to the dose escalation strategy (seven patients at 50 mg/m^2^, three patients at 100 mg/m^2^, and seven patients at 200 mg/m^2^). Two patients discontinued the study due to disease progression before completion of cycle 1; these two patients were excluded from the DLT evaluation.

The median duration of amatuximab treatment was 30 days (36 days at 50 mg/m^2^, 36 days at 100 mg/m^2^, and 29 days at 200 mg/m^2^).

### Safety

Of the 17 patients, 13 (76.5 %) experienced a treatment-related AE. One patient at the 200 mg/m^2^ dose level died due to a treatment-related AE (interstitial lung disease [ILD]). The most common treatment-related AEs were fatigue (five patients, 29.4 %), pyrexia (four patients, 23.5 %), and cytokine release syndrome (three patients, 17.6 %) (Table 2). The grade 3 or higher treatment-related AEs occurred in one patient with a grade 3 cytokine release syndrome at the 50 mg/m^2^ dose level and one patient with a grade 5 ILD at the 200 mg/m^2^ dose level; both were considered DLTs.

**Table 2:** Common treatment-related adverse events (≥10 % frequency)

Dose	50 mg/m^2^ (*n* = 7)			100 mg/m^2^ (*n* = 3)			200 mg/m^2^ (*n* = 7)			Total (*n* = 17)			
Grades	1	2	3	1	2	3	1	2	3	1	2	3	*n* (%)
Fatigue	3	0	0	1	0	0	1	0	0	5	0	0	5 (29.4)
Pyrexia	3	0	0	0	0	0	1	0	0	4	0	0	4 (23.5)
Cytokine release syndrome	0	1	1	0	0	0	0	1	0	0	2	1	3 (17.6)
AST increased	0	2	0	0	0	0	0	0	0	0	2	0	2 (11.8)
Decreased appetite	1	0	0	0	1	0	0	0	0	1	1	0	2 (11.8)
Hot flash	1	0	0	1	0	0	0	0	0	2	0	0	2 (11.8)
Nausea	1	0	0	0	0	0	1	0	0	2	0	0	2 (11.8)
Vomiting	2	0	0	0	0	0	0	0	0	2	0	0	2 (11.8)
Weight decreased	0	1	0	1	0	0	0	0	0	1	1	0	2 (11.8)

*AST* aspartate transaminase

DLTs were evaluated in 15 patients who completed cycle 1. At the initial dose level of 50 mg/m^2^, one of three patients developed a DLT (a grade 3 cytokine release syndrome). Since no additional DLTs occurred in the additional three patients, the dose was escalated first to 100 mg/m^2^ and then to 200 mg/m^2^. At the 200 mg/m^2^ dose level, one of the six patients developed a DLT (a grade 5 ILD). A higher dose than 200 mg/m^2^ was not investigated in this study. As there was only one patient with a DLT at each of the 50 and 200 mg/m^2^ dose levels, the MTD was determined to be 200 mg/m^2^ according to the definition of MTD in this study protocol.

Five of 17 patients (29.4 %) experienced AEIs (Table 3). Common AEIs were cytokine release syndrome (three patients, 17.6 %), pyrexia (two patients, 11.8 %), and hot flush (two patients, 11.8 %). The only grade 3 or higher AEI was a grade 3 cytokine release syndrome (one patient, 5.9 %).

**Table 3:** Adverse events of interest

Dose	50 mg/m^2^ (*n* = 7)			100 mg/m^2^ (*n* = 3)			200 mg/m^2^ (*n* = 7)			Total (*n* = 17)			
Grades	1	2	3	1	2	3	1	2	3	1	2	3	*n* (%)
Total	0	1	1	1	0	0	1	1	0	2	2	1	5 (29.4)
Cytokine release syndrome	0	1	1	0	0	0	0	1	0	0	2	1	3 (17.6)
Hot flush	1	0	0	1	0	0	0	0	0	2	0	0	2 (11.8)
Pyrexia	1	0	0	0	0	0	1	0	0	2	0	0	2 (11.8)
Arthralgia	1	0	0	0	0	0	0	0	0	1	0	0	1 (5.9)
Flushing	0	0	0	0	0	0	1	0	0	1	0	0	1 (5.9)
Nausea	0	0	0	0	0	0	1	0	0	1	0	0	1 (5.9)

### Pharmacokinetics and human anti-chimeric antibody

**Fig. 1:**
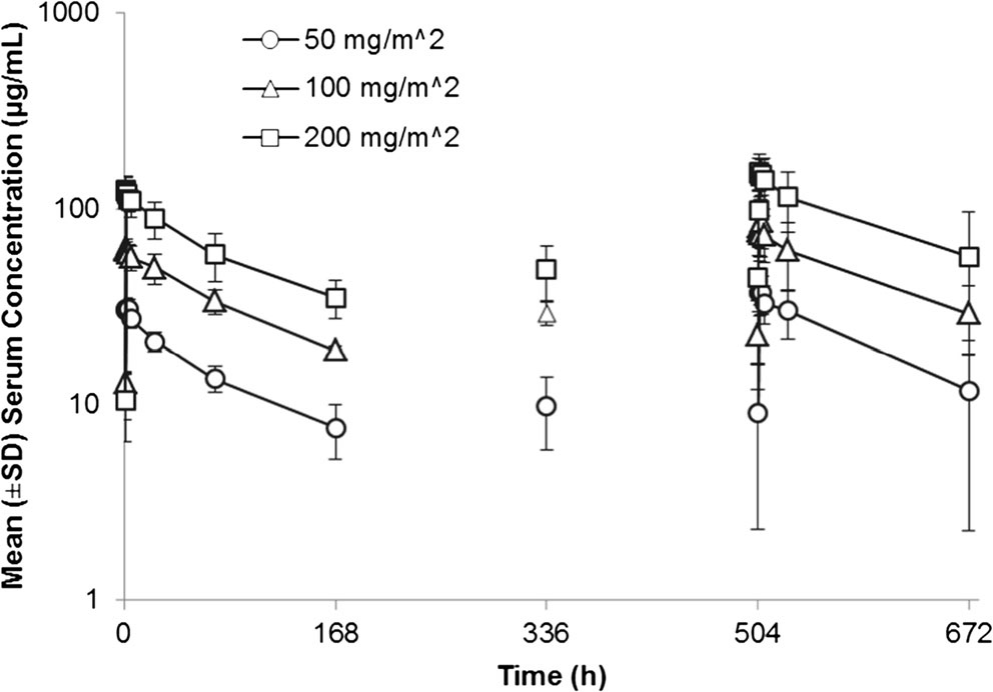
Serum concentration of amatuximab in cycle 1. *n* = 7 in 50 and 200 mg/m^2^, *n* = 3 in 100 mg/m^2^ dose group on cycle 1 day 1. *n* = 5 in 50 mg/m^2^, *n* = 3 in 100 mg/m^2^, *n* = 6 in 200 mg/m^2^ dose group on cycle 1 day 22. *Error bars* show standard deviation. Abbreviations: SD = standard deviation

The serum concentration of amatuximab in cycle 1 is shown in Fig. 1. Amatuximab exhibited a biphasic elimination pattern. Pharmacokinetic parameters on day 1 and day 22 are shown in Table 4. The mean C_max_ on day 1 ranged from 33.2 μg/mL for the 50 mg/m^2^ dose to 133 μg/mL for the 200 mg/m^2^ dose, and the mean AUC_(0-t)_ on day 1 ranged from 2380 to 10300 μg•h/mL. The mean t_1/2_, total clearance, and V_ss_ on day 1 were 92.3 to 108 h, 11.8 to 15.2 mL/h/m^2^, and 1.77 to 2.06 L/m^2^, respectively. The mean C_max_ on day 22 ranged from 40.4 μg/mL to 163 μg/mL, and the mean AUC_(0-t)_ and t_1/2_ values on day 22 ranged from 3210 to 15500 μg•h/mL, and 101 to 154 h, respectively. These results reveal that amatuximab has a low clearance as well as a low distribution volume.

**Table 4:** Pharmacokinetic parameters

Parameter	Dose		
	50 mg/m^2^	100 mg/m^2^	200 mg/m^2^
Day 1	*n* = 7	*n* = 3	*n* = 7
C_max_ (μg/mL)^†^	33.2 ± 3.57	65.1 ± 4.76	133 ± 19.2
t_max_ (h)^‡^	1.73 (0.72–2.72)	1.42 (1.00–1.58)	2.87 (1.52–5.68)
AUC_(0–24)_ (μg•h/mL)^†^	589 ± 49.6	1270 ± 125	2370 ± 403
AUC_(0-t)_ (μg•h/mL)^†^	2380 ± 318	5710 ± 615	10300 ± 2230
AUC_(0-inf)_ (μg•h/mL)^†^	3430 ± 778	8540 ± 588	15800 ± 3440
t_1/2_ (h)^†^	92.3 ± 14.4	104 ± 24.9	108 ± 17.9
CL (mL/h/m^2^)^†^	15.2 ± 2.98	11.8 ± 0.833	13.3 ± 3.46
V_ss_ (L/m^2^)^†^	1.99 ± 0.183	1.77 ± 0.346	2.06 ± 0.465
Day 22	*n* = 5	*n* = 3	*n* = 6
C_max_ (μg/mL)^†^	40.4 ± 6.51	86.1 ± 23.4	163 ± 33.2
t_max_ (h)^‡^	1.270 (0.80–2.28)	2.620 (2.53–2.62)	1.295 (1.12–1.82)
AUC_(0–24)_ (μg•h/mL)^†^	774 ± 184	1650 ± 516	3110 ± 900
AUC_(0-t)_ (μg•h/mL)^†^	3210 ± 1360	8070 ± 2980	15500 ± 6610
t_1/2_ (h)^†^	101 ± 60.9	134 ± 35.9	154 ± 98.3

^†^mean ± standard deviation

^‡^median (min–max)

*AUC* area under the serum concentration-time curve; *AUC*_*(0–24)*_ AUC from zero to 24 h; *AUC*_*(0-t)*_ AUC from zero to the time of last observation; *AUC*_*(0-inf)*_ AUC from zero to infinity; *C*_*max*_ maximum serum concentration; *CL* total clearance; *t*_*1/2*_ terminal half-life; *t*_*max*_ time at which the highest serum drug concentration occurs; *V*_*ss*_ distribution volume at steady state

**Fig. 2:**
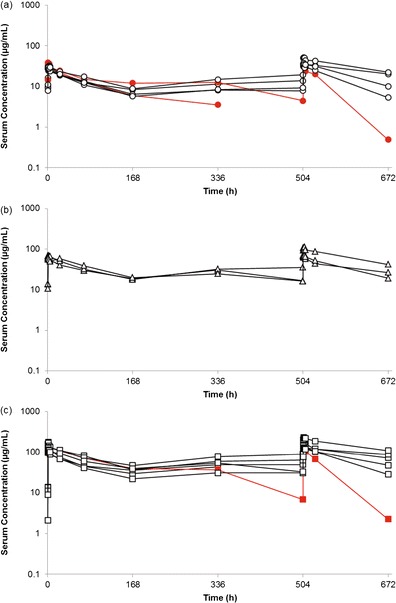
Individual serum concentrations in cycle 1. (**a**) 50 mg/m^2^ dose group (*n =* 7). (**b**) 100 mg/m^2^ dose group (*n =* 3). (**c**) 200 mg/m^2^ dose group (*n* = 7). Red line: HACA-positive patients who showed a decrease in the serum concentration in cycle 1

Eight of 17 patients (47.1 %) had at least 1 positive HACA value during the study. Of these eight HACA-positive patients, one patient was positive for HACA expression prior to receiving the amatuximab infusion and had increased titers during the study. This patient experienced grade 3 cytokine release syndrome and a high titer level of HACA was found. Five patients developed HACA during study treatment and two patients developed HACA at the final sampling point after finishing the study treatment. The occurrence of HACA dropped to 30.8 % (four of 13 patients) when the protocol was amended to mandate premedication with antihistamines and acetaminophen prior to the amatuximab infusion. Five of 17 patients (29.4 %) developed a positive HACA value in cycle 1. A decrease in the serum concentration of amatuximab was observed in three of these five patients (in the remaining two patients, one had low titer levels of HACA and one discontinued study treatment on day 14 due to progressive disease) (Fig. 2). The three patients all experienced a grade 2 or 3 AEI, including grade 3 cytokine release syndrome.

### Efficacy

Based on the investigator assessment, three of 17 patients (17.6 %) had stable disease (SD) and 14 patients (82.4 %) had progressive disease recorded as their best overall response. The durations of SD for the three patients were 47+ (censored), 101, and 217 days.

### Mesothelin immunohistochemistry

Tumor tissue samples from 53 patients were assayed for mesothelin expression by IHC. Tissue samples from 46 patients with solid tumors other than mesothelioma and pancreatic adenocarcinoma were assayed for IHC prior to enrollment because they were required by eligibility criteria to be mesothelin positive. Eighteen of 46 patients were mesothelin positive and nine patients were enrolled in this study. After study completion, we assayed tissue samples obtained from seven patients with mesothelioma and pancreatic adenocarcinoma who provided informed consent for IHC as a biomarker study. Of the seven patients, six patients enrolled were mesothelin positive and one patient not enrolled was mesothelin negative.

The results of mesothelin expression by IHC per tumor type are presented in Table 5. Of 53 patients evaluable for mesothelin expression, 24 patients (45.3 %) were positive for mesothelin expression. Mesothelin expression were observed in mesothelioma (two of two patients, 100.0 %), head and neck cancer (two of two patients, 100.0 %), small intestine cancer (one of one patient, 100.0 %), pancreatic adenocarcinoma (four of five patients, 80.0 %), colorectal cancer (10 of 19 patients, 52.6 %), and biliary cancer (four of eight patients, 50.0 %).

**Table 5:** Mesothelin expression by immunohistochemistry

Tumour type	No. of patients^†^	%
Mesothelioma	2/2	100.0
Head and neck cancer	2/2	100.0
Small intestinal cancer	1/1	100.0
Pancreatic adenocarcinoma	4/5	80.0
Colorectal cancer	10/19	52.6
Biliary cancer	4/8	50.0
Non-small cell lung cancer	1/4	25.0
Other tumour types^‡^	0/12	0.0
Total	24/53	45.3

^†^ Number of patients mesothelin positive / number of patients evaluable for mesothelin expression

^‡^Other tumour types; gastric cancer, small cell lung cancer, sarcoma (two patients, each), orbital abscess, carcinoma of unknown primary, anal cancer, melanoma, esophageal cancer, phyllodes tumour (one patient, each)

## Discussion

In this study, patients with various solid tumor types were screened, with enrollment consisting mainly of patients with colorectal cancer and pancreatic adenocarcinoma. The MTD was determined to be 200 mg/m^2^. A higher dose than 200 mg/m^2^ was not investigated in this study because 400 mg/m^2^ was judged as intolerable due to two DLTs (elevation of ALT/AST and serum sickness) in the previous US phase I study [13].

The most common treatment-related AEs were fatigue and pyrexia, which were grade 1 and easily managed. The grade 3 or higher treatment-related AEs were cytokine release syndrome (grade 3) and ILD (grade 5), which occurred in one patient each. There were no apparent dose-related trends in frequency or severity of treatment-related AEs. Five patients experienced AEIs; however, all AEIs were manageable. These findings are consistent with the US phase I single agent amatuximab study [13]. Two patients experienced DLTs in this study (cytokine release syndrome and ILD each), whereas three patients experienced DLTs in the US phase I study (deep venous thrombosis at 100 mg/m^2^, elevation of ALT/AST and serum sickness at 400 mg/m^2^). In this study, a grade 2 AST increased was observed in two patients; moreover, events of grade 2 and 3 cytokine release syndrome were observed in two patients and one patient, respectively.

The first DLT involving grade 3 cytokine release syndrome occurred during the fourth infusion of amatuximab (on day 22) in the first patient at the 50 mg/m^2^ dose level. No premedication to prevent allergic reaction were administered to this patient. The patient experienced symptoms, including dyspnea, within minutes after starting the amatuximab infusion. All symptoms were resolved by stopping the infusion. The amatuximab infusion was resumed at a lower rate (1 mg/min); however, dyspnea, chills, and shivering subsequently appeared with marked wet rales of lung. Consequently, the amatuximab infusion was discontinued. These symptoms disappeared rapidly with antihistamines and corticosteroid administration, and this event of cytokine release syndrome was resolved on the following day (day 23). Hypotension did not occur with this adverse event. No subsequent amatuximab infusions were administered to this patient, who was then withdrawn from the study. Because grade 2 cytokine release syndrome with chill and shivering also occurred in a fourth patient at the 50 mg/m^2^ dose level, the study protocol was amended to make premedication of antihistamines and acetaminophen mandatory. After the protocol amendment, no AEI of grade 3 or higher occurred and the frequency of AEIs decreased. Consequently, premedication would be required in the next phase study as well.

The second DLT was a grade 5 ILD in a patient with pleural mesothelioma. After the fourth infusion of amatuximab (final infusion in the patient) on day 22, a grade 2 ILD occurred on day 27, complicated by a grade 3 pneumonia with no causal relationship to amatuximab. Although the symptoms of pneumonia were improved by the antibacterial agent on day 33, the patient died due to the ILD on day 60. Considering the time course from amatuximab infusion to the onset of the event, a causal relationship with amatuximab infusion could not be ruled out with certainty; therefore, it was considered to be probably related. Given the clinical findings, the direct cause of death appeared to be respiratory failure due to aggravation of interstitial pneumonia rather than the underlying pleural mesothelioma. Although no other patients experienced AEs related to ILD in either this study or the US phase I study [13], careful attention should be paid to ILD.

**Fig. 3:**
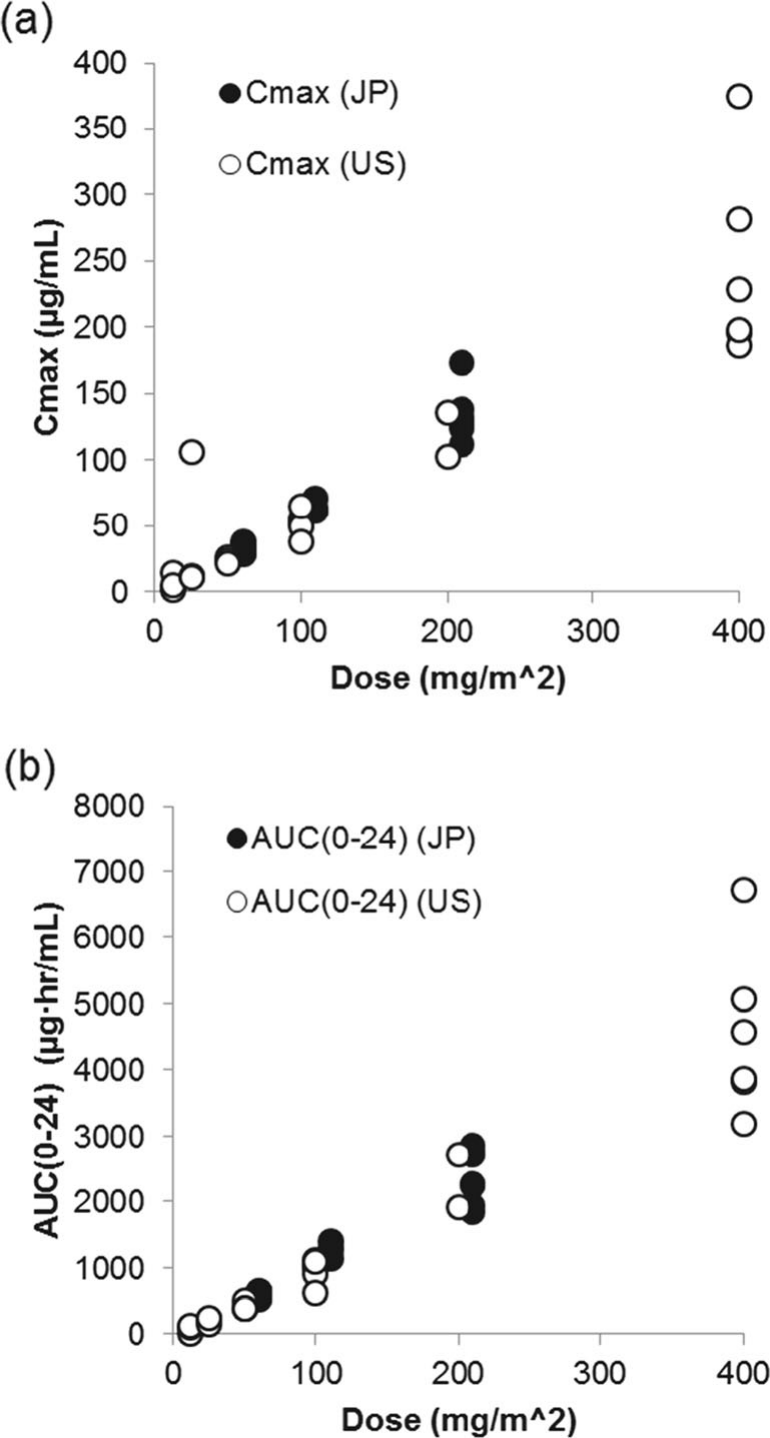
Relationship between dose and pharmacokinetic parameters of C_max_ and AUC_(0–24)_ on cycle 1 day 1. (**a**) C_max_. (**b**) AUC_(0–24)_. Abbreviations: AUC = area under the concentration curve; AUC_(0–24)_ = AUC from zero to 24 h; C_max_ = maximum serum concentration; JP = Japan; US = United States

Pharmacokinetic analysis revealed that amatuximab exhibited a biphasic distribution and was gradually eliminated. The pharmacokinetic profile of amatuximab was characterized by low clearance and a low distribution volume, which is similar to the pharmacokinetic profiles exhibited by other anti-receptor antibodies [15]. Fig. 3 shows the relationship between dose and the pharmacokinetic parameters of C_max_ and AUC_(0–24)_ on cycle 1 day 1. The results demonstrate that the C_max_ and AUC values increased in an almost dose-proportional manner within a dose range of 50 to 200 mg/m^2^. In addition, these data show that the pharmacokinetic profile of amatuximab in Japanese patients is similar to that in the US patients.

In this study, eight of 17 treated patients (47.1 %) developed HACA. The frequency of patients developing HACA-positivity in this study is high as compared to seven of 24 treated patients (29.2 %) in the US phase I study [13]. However, the occurrence of HACA in this study dropped to 30.8 % (four of 13 patients with premedication) as compared to 100.0 % (four of four patients without premedication). Furthermore, the four HACA-positive patients with premedication included one patient whose HACA titer level was low and two patients who developed HACA at the final sampling point after finishing study treatment and premedications. This means that only one of the 13 patients developed HACA with high titer levels during the premedication treatment period. Thus, premedication tends to decrease expression of HACA, although the mechanism behind how the HACA expression is controlled by premedication of antihistamines and acetaminophen is unknown.

Although there was no objective tumor response to amatuximab, three patients (17.6 %) experienced SD, most of whom had been heavily pre-treated. There were two colorectal cancer patients and one pancreatic adenocarcinoma patient with periods of durable SD by amatuximab monotherapy, but the numbers were too small to be meaningful. Results of a single arm phase II study in patients with pleural mesothelioma indicate that a median overall survival of 14.8 months with a third of the patients alive at the time of analysis is suggestive of antitumor activity arising from the combination of amatuximab plus pemetrexed/cisplatin [16]. A randomized, placebo-controlled study is planned to investigate the survival benefit of this combination.

Mesothelin expression was observed by IHC in multiple patients with mesothelioma, head and neck, pancreatic adenocarcinoma, colorectal cancer, and biliary cancer. The frequencies of patients with mesothelin expression were high in mesothelioma and pancreatic adenocarcinoma, consistent with prior reports [6–8]. The relationship between efficacy and mesothelin expression is unclear, since apparent tumor regression could not be observed in this study. However, considering the mode of action by amatuximab, colorectal cancer and biliary cancer, which were frequently reported to be mesothelin positive in this study, can be potential targets in the future clinical development of amatuximab. As a predictive biomarker for amatuximab response was not identified in this study, further biomarker research including mesothelin expression should be done in future clinical study.

In conclusion, the MTD of this study was determined to be 200 mg/m^2^. However, the actual MTD of amatuximab might be higher than 200 mg/m^2^, since dosing above 200 mg/m^2^ was not investigated in this study. Amatuximab was generally well tolerated at the 50, 100, or 200 mg/m^2^ dose level in patients with solid tumors. Although there were no objective responses, disease stabilization was observed in some patients. The efficacy of amatuximab in combination with cytotoxic drugs is to be investigated in other studies. Amatuximab exhibited a biphasic distribution and was gradually eliminated. The C_max_ and AUC values on cycle 1 day 1 increased in an almost dose-proportional manner within a dose range of 50 to 200 mg/m^2^. In addition, the pharmacokinetic profile of amatuximab in Japanese patients is similar to that in the US patients.
